# Prediction of enhancer-promoter interactions via natural language processing

**DOI:** 10.1186/s12864-018-4459-6

**Published:** 2018-05-09

**Authors:** Wanwen Zeng, Mengmeng Wu, Rui Jiang

**Affiliations:** 1 MOE Key Laboratory of Bioinformatics; Bioinformatics Division and Center for Synthetic & Systems Biology, Beijing, 100084 China; 2 Department of Automation, Tsinghua University, Beijing, 100084 China; 3 Department of Computer Science, Tsinghua University, Beijing, 100084 China

**Keywords:** Enhancer-promoter interactions, Three-dimensinal interactions, Natural language processing, Unsupervised learning

## Abstract

**Background:**

Precise identification of three-dimensional genome organization, especially enhancer-promoter interactions (EPIs), is important to deciphering gene regulation, cell differentiation and disease mechanisms. Currently, it is a challenging task to distinguish true interactions from other nearby non-interacting ones since the power of traditional experimental methods is limited due to low resolution or low throughput.

**Results:**

We propose a novel computational framework EP2vec to assay three-dimensional genomic interactions. We first extract sequence embedding features, defined as fixed-length vector representations learned from variable-length sequences using an unsupervised deep learning method in natural language processing. Then, we train a classifier to predict EPIs using the learned representations in supervised way. Experimental results demonstrate that EP2vec obtains F1 scores ranging from 0.841~ 0.933 on different datasets, which outperforms existing methods. We prove the robustness of sequence embedding features by carrying out sensitivity analysis. Besides, we identify motifs that represent cell line-specific information through analysis of the learned sequence embedding features by adopting attention mechanism. Last, we show that even superior performance with F1 scores 0.889~ 0.940 can be achieved by combining sequence embedding features and experimental features.

**Conclusions:**

EP2vec sheds light on feature extraction for DNA sequences of arbitrary lengths and provides a powerful approach for EPIs identification.

**Electronic supplementary material:**

The online version of this article (10.1186/s12864-018-4459-6) contains supplementary material, which is available to authorized users.

## Background

One of the major discoveries in recent years is that non-coding DNAs are not “junk”. On the contrary, they fulfill a wide variety of crucial biological roles involving regulatory and signaling functions [1]. Enhancer is one of the most important noncoding elements that has a central role in controlling gene expression [2]. Recent studies have shown that noncoding single nucleotide polymorphisms (SNPs) that are associated with risk for numerous common diseases through genome-wide association studies (GWAS), frequently lie in cell line-specific enhancers [3, 4]. These GWAS SNPs are hard to interpret because we are unaware of how non-coding SNPs affect gene expression and disease transmission through the complicated regulatory relationship [5]. We can improve understanding of disease mechanisms if enhancers are accurately linked to the promoters/genes they regulate. For example, Guo et al. [6] identified mechanism of GWAS risk SNP rs7463708 in promoting prostate transformation. This SNP is located in the enhancer of long noncoding RNA (lncRNA) PCAT1 and significantly upregulates PCAT1 expression. PCAT1 interacts with the enhancers of prostate cancer genes GNMT and DHCR24, and in turn promotes prostate tumorigenesis. Thus, the identification of true three-dimensional (3D) genome organization, especially EPIs across different cell lines constitutes important steps towards understanding of gene regulation, cell differentiation and disease mechanisms.

However, there are enormous technical challenges to obtain these 3D interactions in the entire genome. Chromosome conformation capture-based (3C) methods [7], including 4C [8] and 5C [9] have been developed to detect physical contacts in the 3D space but fail to capture whole genome interactions. Chromatin Interaction Analysis by Paired-End Tag Sequencing (ChIA-PET) [10] allows genome-wide measurements but is restricted to interactions mediated by a preselected protein of interest. The method of Hi-C [11] allows the genome-wide detections of interactions but its current resolution is not high enough (~ 10 kb) to capture individual EPIs. All these traditional experimental approaches for detecting 3D genome interactions remain time-consuming and noisy, motivating the development of computational approaches.

To bridge this growing gap between low-resolution experiments and high-resolution EPIs, some computational methods have been established, which mainly fall into two classes. One class is based on experimental features. For instance, IM-PET [12], RIPPLE [13], TargetFinder [14] and EpiTensor [15], aim to predict 3D genomic interactions in different cell lines by integrating numerous one-dimensional (1D) local chromatin states including genomic and epigenomic data. Among them, TargetFinder is the state-of-the-art computational method to identify true EPIs by collecting experimental data sets including histone modifications, TF binding, chromatin accessibility and gene expressions. The other class is based on sequence information only, which is represented by SPEID [16]. SPEID takes advantage of a convolutional Long Short-Term Memory (LSTM) network to learn the feature representation from input sequences automatically and can reliably predict EPIs.

Existing 3D genomic interaction prediction methods fail to exploit sequence information except SPEID. At the meantime, there are many inspiring methods for 1D chromatin states prediction [17, 18], including gkmSVM for enhancer prediction [19], DeepSEA for epigenomic state prediction [20] and DeepBind for DNA/RNA-binding proteins prediction [21], which extract sequence features and yield high performance. gkmSVM transforms variable-length sequences to fixed-length *k*-mer features to classify input DNA sequences. *k*-mer features are an unbiased, complete set of sequence features defined on arbitrary-length DNA sequences but lose the contextual information between adjacent *k*-mers. DeepSEA and DeepBind take advantage of powerful convolutional neural networks (CNN) but they require fixed-length sequences as input, which is also a limit for SPEID. Since DNA sequences are in variable length and contextual information is important for understanding the function of whole sequence, how to transform a variable-length sequence into a fixed-length vector representation conserving the context information remains challenging and crucial for improving sequence-based prediction methods.

It is well-known that learning a good representation of input data is an important task in machine learning. There is an analogous problem in natural language processing, which is to learn an embedding vector for a sentence, that is essentially, to train a model that is able to automatically transform a sentence to a vector and encodes its semantic meaning. Paragraph Vector [22] successfully solves the problem by mapping texts into a unified vector representation, and generates embedding representation which can be further used for different applications [23], such as machine translation [24], sentiment analysis [22], and information retrieval [25].

Inspired by the idea of sentence embedding, we present a novel 3D interactions prediction method, named EP2vec, in this paper. First, we utilize an unsupervised deep learning method, namely Paragraph Vector, to learn sequence embedding features. Concretely, we embed the enhancer sequences and promoter sequences into a vector space separately, and then every sequence can be represented as a vector, namely the sequence embedding features. Then, EP2vec uses the resulted features for subsequent classification of EPIs through supervised learning. Our experiments prove that we are able to accurately predict EPIs using only the sequence embedding features, which outperforms other existing computational methods. In addition, by combining both sequence embedding features and experimental features, we can further improve performance, which indicates sequence embedding features and experimental features are complementary to each other. Furthermore, by applying attention mechanism, we successfully interpret the meaning of sequence embedding features and find motifs that represent cell line information. The source code to implement EP2vec can be downloaded from https://github.com/wanwenzeng/ep2vec.

## Methods

### Datasets

The majority of our datasets were adapted from TargetFinder. Promoter and enhancer regions were identified using ENCODE Segway [26] and ChromHMM [27] annotations for K562, GM12878, HeLa-S3, and HUVEC cell lines, and using Roadmap [28] Epigenomics ChromHMM annotations for NHEK and IMR90 cell lines. Since EPIs could only happen between active enhancers and promoters, we used the full set of all enhancers and promoters as external resources to perform unsupervised feature extraction which would be elaborated in the next section. The total number of enhancers and the number of promoters for each cell line are reported in Table 1. The length distributions of enhancers and promoters in six cell lines are shown in Additional file 1: Figures S1 and S2.

**Table 1:** Details of each cell line dataset. The enhancers (or promoters) column indicates the number of all known active enhancers (or promoters) for each cell line, which are used for unsupervised feature learning for enhancer (or promoter) sequences

Dataset	enhancers	promoters	true EPIs	false EPIs
K562	82806	8196	1977	1975
IMR90	108996	5253	1254	1250
GM12878	100036	8453	2113	2110
HUVEC	65358	8180	1524	1520
HeLa-S3	103460	7794	1740	1740
NHEK	144302	5254	1291	1280
FANTOM	43011	49620	61542	61542

To focus on distal interactions, enhancers closer than 10 kb to the nearest promoter were discarded. Using GENCODE [29] version 19 annotations and RNA-seq data from ENCODE, promoters were reserved if actively transcribed (mean FPKM > 0.3 [30] with irreproducible discovery rate < 0.1 [31]) in each cell line. Positive EPIs were annotated using high-resolution genome-wide Hi-C data [32]. These EPIs were assigned to one of five bins based on the distance between the enhancer and the promoter, such that each bin had the same number of interactions. Negative pairs were assigned to their corresponding distance bin and then subsampled within each bin, using one negative per positive. The number of positive or negative samples for each cell line is reported in Table 1.

In addition, we also collected a dataset from FANTOM5 project [4]. The FANTOM5 consortium extracted RNA transcripts from a multitude of different primary cells and tissues using the Cap Analysis of Gene Expression (CAGE) experiment. Because active enhancer regions were transcribed, they identified a distinct bidirectional CAGE pattern which could predict enhancer regions based on CAGE data not associated with promoters. The transcribed enhancer atlas held around 40,000 transcribed enhancers across the human body, which they called permissive enhancers. We collected the permissive enhancers and RefSeq promoters. Using statistical methods, FANTOM5 defined some enhancer-promoter interactions, which we considered as positive samples. Negative samples were generated as random pairs of enhancers and promoters based on the distance distribution of the positive samples.

### Workflow of EP2vec

**Fig. 1:**
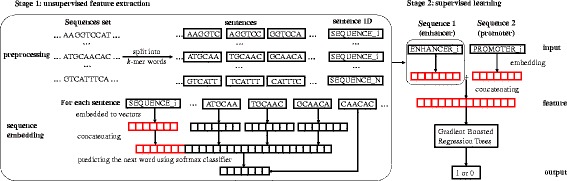
The two-stage workflow of EP2vec. Stage 1 of EP2vec is unsupervised feature extraction which transforms enhancer sequences and promoter sequences in a cell line into sequence embedding features separately. Given a set of all known enhancers or promoters in a cell line, we first split all the sequences into *k*-mer words with stride *s* = 1 and assign a unique ID to each of them. Regarding the preprocessed sequences as sentences, we embed each sentence to a vector by Paragraph Vector. Concretely, we use vectors of words in a context with the sentence vector to predict the next word in the context using softmax classifier. After training converges, we get embedding vectors for words and all sentences, where the vectors for sentences are exactly the sequence embedding features that we need. Note that in sentence ID, SEQUENCE is a placeholder for ENHANCER or PROMOTER, and is the total number of enhancers or promoters in a cell line. Stage 2 is supervised learning for predicting EPIs. Given a pair of sequences, namely an enhancer sequence and a promoter sequence, we represent the two sequences using the pre-trained vectors and then concatenate them to obtain the feature representation. Lastly, we train a Gradient Boosted Gradient Trees classifier to predict whether this pair is a true EPI

The workflow of EP2vec contained two stages including the unsupervised feature extraction and supervised learning (Fig. 1). Sequences of active regulatory elements in a specific cell line have cell line-specific regulatory information. Hence, EP2vec could use unsupervised methods to extract useful information from the sequences set, which would benefit subsequent tasks such as EPIs prediction. EP2vec regarded DNA sequences as sentences with *k*-mers as words, and learned effective representations of these sequences based on the co-occurrence statistics of *k*-mers.

Stage 1 of EP2vec was unsupervised feature extraction which transforms enhancer sequences and promoter sequences in a cell line into sequence embedding features separately. Given a set of all known enhancers or promoters in a cell line, we first split all the sequences into *k*-mer words with stride s = 1 and assign a unique ID to each of them. Regarding the preprocessed sequences as sentences, we embedded each sentence to a vector by Paragraph Vector. Concretely, we used vectors of words in a context with the sentence vector to predict the next word in the context using softmax classifier. After training converges, we got embedding vectors for words and all sentences, where the vectors for sentences were exactly the sequence embedding features that we needed. Stage 2 is supervised learning for predicting EPIs. Given a pair of sequences, namely an enhancer sequence and a promoter sequence, we represented the two sequences using the pre-trained vectors and then concatenated them to obtain the feature representation. Lastly, we trained a Gradient Boosted Regression Trees classifier (GBRT) to predict whether this pair was a true EPI.

### Feature extraction

In this section, we will illustrate how to apply Paragraph Vector to learn fixed-length feature representations from variable-length DNA sequences in Stage 1.

First, given a set of all known enhancers or promoters in a cell line, we assigned a unique ID for each sequence, and split it into *k*-mers. *k*-mers were split along a sequence using sliding window with stride *s*, meaning that two adjacent *k*-mers had a distance of *s* bps. Thus, in general, a sequence with *L* bps will be split into $floor\left[\frac{L-k}{s}\right]+1$ *k*-mers. For example, we could split “ATGCAACAC” into four 6-mers with stride s = 1 as “ATGCAA”, “TGCAAC”, “GCAACA” and “CAACAC” with ID “SEQEUNCE_i” (Fig. 1). From now on, we regarded the split enhancers or promoters as sentences and the *k*-mers as words. Note that the vocabulary size of *k*-mers was 4^*k*^.

Second, each sentence was mapped to a unique vector in a *d*-dimensional vector space, where *d* was the embedding dimension. Each word was also mapped to a unique vector in the same space. The basic training algorithms were greedy in nature, we followed general pratics to initialize all these vectors at random before training. For example, the sentence “SEQEUNCE_i” was mapped to a *d*-dimensional vector *x*_*i*_ ∈ *ℝ*^*d*^, with each component initialized by a random value. Similarly, *k*-mers “ATGCAA”, “TGCAAC”, “GCAACA” and “CAACAC”, which were indexed as *c*_*i*, 1_, *c*_*i*, 2_, *c*_*i*, 3_, *c*_*i*, 4_ ∈ [1, 4^*k*^] in the *k*-mer vocabulary, were also mapped to four vectors ${w}^{c_{i,1}},{w}^{c_{i,2}},{w}^{c_{i,3}},{w}^{c_{i,4}}\in {\mathbb{R}}^d$ with random initialization.

Third, we trained all these sentence vectors and word vectors by constructing the training loss function. In detail, we predicted the next word of a context in a sentence, by concatenating these vectors of words in this context and the sentence vector as predictive features. Since the vocabulary size was 4^*k*^, the next word had 4^*k*^ possibilities. Generally speaking, the context has a fixed window length *m* and is sampled from the sentence in a sliding window fashion. For example, as shown in Fig. 1, we set the window length *m* = 3, and used the concatenated vectors of “ATGCAA”, “TGCAAC”, “GCAACA” and “SEQUENCE_i” to predicted the next word “CAACAC” by a 4096-way classification. Note that the sentence vector was shared across all contexts generated from this single sentence, while the word vector for one single *k*-mer was shared across all sentences.

More formally, given *N* sequences represented in *N* vectors *x*_1_, *x*_2_, …, *x*_*N*_. The *i*-th sequence contained *T*_*i*_ words represented in vectors ${w}^{c_{i,1}},{w}^{c_{i,2}},\dots, {w}^{c_{i,{T}_i}}$, the objective of the model was to maximize the average log probability

$$ \max \sum \limits_{i=1}^N\frac{1}{T_i-m}\sum \limits_{t=1}^{T_i-m}\log p\left({c}_{i,t+m}|{w}^{c_{i,t}},\cdots, {w}^{c_{i,t+m-1}},{x}_i\right). $$

The prediction task was typically accomplished via a multiclass classifier, such as softmax classifier, which could be formulated as

$$ p\left({c}_{i,t+m}|{w}^{c_{i,t}},\cdots, {w}^{c_{i,t+m-1}},{x}_i\right)=\frac{e^{y_{c_{i,t+m}}}}{\sum \limits_j{e}^{y_j}}. $$

Here, *j* ∈ [1, 4^*k*^] was an index to an output words, and *y*_*j*_ was the corresponding component of the un-normalized log-probability $y\in {\mathbb{R}}^{4^k}$ computed by

$$ y=b+ Uh, $$

where $U\in {\mathbb{R}}^{4^k\times \left(m+1\right)d}$ and $b\in {\mathbb{R}}^{4^k}$ are the softmax parameters, while $h=\left({w}^{c_{i,t}},\cdots, {w}^{c_{i,t+m-1}},{x}_i\right)\in {\mathbb{R}}^{\left(m+1\right)d}$ was the concatenation of the *m* word vectors and the sentence vector.

The *N* sentence vectors and 4^*k*^word vectors were trained using stochastic gradient descent (SGD) together with the softmax parameters *U* and *b*, where the gradient was obtained via back propagation [33]. At every step of SGD, one could sample a fixed-length context from a random sentence, compute the error gradient and use the gradient to update the parameters in our model. In practice, hierarchical softmax [34–36] was preferred to softmax for fast training. In our study, the structure of the hierarchical softmax was a binary Huffman tree, where short codes were assigned to frequent words. This was a good speedup trick because common words were accessed quickly. This use of binary Huffman code for the hierarchical softmax was the same as Mikolov et al. [36].

After the training converges, words with similar meanings were expected to be mapped to adjacent positions in the vector space and the sentence vectors could be used as features for the sentence. In fact, the sentence vectors learned by the model were exactly the sequence embedding features which captured the sequence contextual information. Note that, we trained sequence embedding features for enhancers and promoters separately. We implemented these feature extraction based on the GENSIM packages [37].

### Model training

In this section, we proceeded to interpret Stage 2 of EP2vec workflow (Fig. 1), namely supervised learning for EPIs classification. For each pair of an enhancer and a promoter, we first concatenated the sequence embedding features of the two sequences as the final features. Then based on this feature representation, we trained a GBRT classifier to predict the binary label, i.e., whether this pair was a true EPI. GBRT was a classifier which used decision trees as weak estimators and combines several weak estimators into ensemble as a single model, in a stage-wise fashion. The tree ensemble model was a set of classification and regression trees (CART). The prediction scores of each individual tree were summed up to get the final score.

GBRT performed gradient descent algorithm for the objective function for the binary classification of EPIs, and its performance mainly depended on three hyper-parameters: learning rate *α*, number of trees *n*, and tree-depth D. Smaller learning rates tended to result in better accuracy but require more iterations. Tree-depth D controlled the size of each decision tree. To yield the best performance, we figured out best hyper-parameter setting *α* = 1*e* − 3, *n* = 4000, *D* = 25, using grid search strategy. More details about training of GBRT could be found in the online codes.

### Model evaluation

To examine the performance of EP2vec in predicting EPIs in specific cell line, we performed the stratified 10-fold cross-validation experiment in all datasets. We randomly partitioned training data into ten equal sized subsets and each subset contained roughly the same proportions of the two lines of class labels. One of the ten subsets was used for testing the model, and the remaining nine were used as training data. This validation process was repeated ten times, with each of the ten subsets used exactly once as test data.

We calculated F1 scores for each cross-validation, which considered both the precision p and the recall r of the test. Precision p is the number of correct positive results divided by the number of all positive results, and recall r is the number of correct positive results divided by the number of positive results that should have been returned. The F1 score could be interpreted as the harmonic mean of the precision and recall, as F1 = 2*rp*/(*r* + *p*), which reaches its best value at 1 and worst at 0.

We compared the performance of EP2vec and several other baseline methods, including TargentFinder, gkmSVM, SPEID. We directly used the source codes their authors published online. TargetFinder definde three training sets. The first set included features for the enhancer and promoter only (E/P). The second set included features for an extended enhancer (using 3 kb of flanking sequence) and a non-extended promoter (EE/P). The last set included enhancers and promoters plus the window between them (E/P/W), which were up to thousands of base pairs. Since the performance of TargetFinder on the last set was consistently better than other two sets according to their publication, we only evaluated this method on the E/P/W set. For gkmSVM, we need to first transformed a pair of two sequences (enhancer and promoter) into a single sequence by concatenating them, and then used it as input for gkmSVM.

### Attention mechanism

Not all words contribute equally to the representation of the sentence meaning. Hence, we introduced attention mechanism to find out such critical words that were most important to the meaning of the sentence. Considering the *k*-mer words as motifs, we essentially aimed to find motifs that contribute more to the vector representation of the enhancer/promoter. Take the *i*-th enhancer *x*_*i*_ as example, it contained *T*_*i*_ words ${w}^{c_{i,1}},{w}^{c_{i,2}},\dots, {w}^{c_{i,{T}_i}}$. We measured the importance of each word by computing similarity between word vector ${w}^{c_{i,t}}$ and sentence vector *x*_*i*_ and got a normalized importance weight *α*_*it*_ through a softmax function, as

$$ {\alpha}_{it}=\frac{\exp \left({x}_i^T{w}^{c_{i,t}}\right)}{\sum \limits_j\exp \left({x}_i^T{w}^{c_{i,j}}\right)}. $$

Therefore, every word in the sentence had a weight representing its importance to the sentence. In order to validate that our model was able to select informative words in a sentence, we visualized the high-weight words. In detail, two sets of informative *k*-mers were obtained by picking out the most important words for enhancer and promoter respectively, in every sentence with positive label. Then we performed motif enrichment analysis using CentriMo [38] to compare these words against known motifs in the HOCOMOCO v9 dataset [39], and drew out top enriched motifs with sequence logo [40].

## Results

### Computational performance

To consolidate the importance of our work, we compare the performance of EP2vec against other three typical baseline methods, including TargetFinder, gkmSVM and SPEID. TargetFinder is based on experimental features obtained from biological sequencing experiments, and gkmSVM is based on *k*-mer features and SVM classifiers. SPEID is based on deep learning which uses LSTMs with sequence data to predict EPIs.

They all have their own advantages and disadvantages. (1) For TargetFinder, experimental features are rich of cell line-specific predictive information, but they are expensive and time-consuming to acquire. Besides, for some cell lines, the dimension of accessible experimental features is limited due to lack of biological experiments. (2) For gkmSVM, *k*-mer features are an unbiased, general, complete set of sequence features defined on arbitrary-length sequences. However, the *k*-mers can only capture local motif patterns because they only use the *k*-mer counts information without making full use of context information or co-occurrence information of *k*-mers. (3) For SPEID, LSTM is a powerful supervised deep learning technique which is able to capture long-range dependencies. Nonetheless, deep learning methods often have millions of parameters to learn in the training process which takes a long time, and special attention should be put on fine-tuning the network. Usually, it takes time to optimize the network structure for a specific dataset, but this optimal structure may be not applicable to other datasets due to overfitting problems.

Our paper proposes an innovative approach to represent a DNA sequence (or a pair of two DNA sequences) in a fixed-length vector, namely sequence embedding features, using the unsupervised method Paragraph Vector. The training of sequence embedding features utilizes the global statistics information of *k*-mers, and hence our features form a potentially better presentation for DNA sequences. Specifically, for EP2vec, we set *k* = 6, the stride *s* = 1, the context window size *m* = 20, and the embedding dimension *d* = 100. We report the F1 score statistics of the four methods in 10-fold cross-validation for each dataset in Table 2. In addition, we also calculate area under the Receiver Operating Characteristic curve (auROC) score (Additional file 1: Table S1) and area under the Precision Recall curve (auPRC) score (Additional file 1: Table S2).

**Table 2:** The mean values and the standard deviations of F1 scores for EP2vec and other three baseline methods in 10-fold cross-validation experiments**.** For FANTOM dataset, we do not evaluate TargetFinder due to lack of experimental features, and we do not evaluate SPEID since it is extremely time-consuming to run 10-fold cross validation of SPEID on so many samples

Dataset	EP2vec	TargetFinder	gkmSVM	SPEID
K562	0.882 (0.019)	0.881 (0.014)	0.821 (0.018)	0.846 (0.024)
IMR90	0.872 (0.020)	0.863 (0.017)	0.749 (0.026)	0.825 (0.032)
GM12878	0.867 (0.014)	0.844 (0.010)	0.779 (0.015)	0.809 (0.018)
HUVEC	0.875 (0.024)	0.878 (0.022)	0.731 (0.028)	0.809 (0.023)
HeLa-S3	0.920 (0.013)	0.913 (0.014)	0.822 (0.021)	0.888 (0.023)
NHEK	0.933 (0.015)	0.922 (0.018)	0.800 (0.024)	0.900 (0.019)
FANTOM	0.841(0.004)	/	0.803(0.017)	/

The results in Table 2 show that EP2vec is slightly better than TargetFinder and significantly outperforms the other two sequence-based methods, namely gkmSVM and SPEID. For example, in the GM12878 cell line dataset, the average F1 scores of EP2vec, TargetFinder (on E/P/W), gkmSVM and SPEID are 0.867, 0.844, 0.779 and 0.809, respectively. On the whole, the F1 scores for six cell line datasets of the above four methods ranges from 0.867~ 0.933, 0.844~ 0.922, 0.731~ 0.822, 0.809~ 0.900, respectively. We are convinced that the sequence embedding features learned by EP2vec is comparable to experimental features and has superiority over the other two computational sequence features, because we are able to capture the global context information of DNA sequences.

### Sensitivity analysis

The goal of EP2vec is to capture global sequence information. In our approach, we must split sequences into words using a sliding window fashion to form sentences from which we could extract fixed-length embedding features. To evaluate the stability of EP2vec, we carry out sensitivity analysis for hyper-parameters including *k*, the stride *s* and the embedding dimension *d*.

**Fig. 2:**
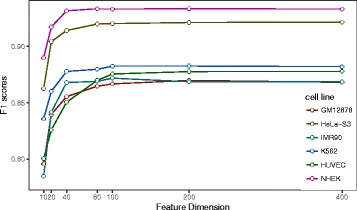
The F1 scores of different embedding dimensions. As the embedding dimensions increase, the performance increses. And embedding dimension *d* = 100 is sufficient to obtain the near-optimal performances in all these datasets

As shown in Fig. 2, we find that when the embedding dimension d decreases, our model degrades slightly. For example, the F1 score of EP2vec on HUVEC dataset is 0.875 when *d* = 100. Setting *d* = 10 and retaining the other hyper-parameters unchanged, we find the F1 score decreases to 0.800. In general, the performance improves with the increase of embedding feature dimension. We note that although the mean F1 scores are not similar across different cell lines, 100 is the common choice of embedding dimension to obtain the near-optimal performances for all datasets. Lower but acceptable performance requires embedding dimension of 40 in NHEK, IMR90 and K562 while 80 in the other cell lines.

Furthermore, we explore the performance of different settings of the model hyper-parameters including *k,* the stride *s* and the embedding dimension *d*. The sensitivity analysis of these hyper-parameters is shown in Additional file 1: Tables S3-S5. These results indicate that EP2vec is robust to all the three hyper-parameters and successful in capturing the information of whole sentences.

### Visualizing motifs by attention mechanism

**Fig. 3:**
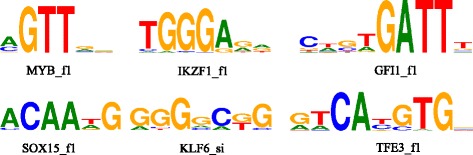
The enriched motifs in HUVEC and K562. MYB_f1, IKZF1_f1, GFI1_f1 and SOX15_f1 are enriched in HUVEC. KLF6_si and TFE3_f1 are enriched in K562

In order to interpret that our model is able to detect informative *k*-mers or motifs in a sequence, we visualize *k*-mers with high weights selected using the attention mechanism for K562 and HUVEC in Fig. 3. We consider the most informative *k*-mers as sequence motifs that determine sequence function. Consequently, we calculate the most informative *k*-mers in positive samples and present the top enriched known motifs in enhancers and promoters (Additional file 1: Tables S6 and S7).

For example, HUVECs are cells derived from the endothelium of veins from the umbilical cord and are reported to play an important role in hematopoiesis. Among the top five enriched motifs in HUVEC, MYB_f1, GFI1_f1, IKZF1_f1 and SOX15_f1 present some clues to HUVEC cell line-specific information. MYB_f1 will bind to MYB, which plays an essential role in the regulation of hematopoiesis. MYB may be aberrantly expressed or rearranged or undergo translocation in leukemias and lymphomas, and is considered to be an oncogene [41]. GFI1_f1 will bind to GFI1, which functions as a transcriptional repressor. This TF plays a role in diverse developmental contexts, including hematopoiesis and oncogenesis. It functions as part of a complex along with other cofactors to control histone modifications that lead to silencing of the target gene promoters [42]. IKZF1_f1 will bind to IKZF1, which belongs to the family of zinc-finger DNA-binding proteins associated with chromatin remodeling. Overexpression of some dominant-negative isoforms have been associated with B-cell malignancies, such as acute lymphoblastic leukemia [43]. SOX15_f1 will bind to SOX15, which is involved in the regulation of embryonic development and in the determination of the cell fate [44]. All of these top enriched motifs in HUVEC are experimentally proved to be related with hematopoiesis or other similar functions, which indicates that we successfully find informative motifs through applying attention mechanism in EP2vec.

As another example, K562 cells are of the erythroleukemia type, and the line is derived from a 53-year-old female chronic myelogenous leukemia patient in blast crisis. The top two enriched motifs in K562 is KLF6_si and TFE3_f1, which also give evidence to K562 specific information. KLF6_si will binding to KLF6. The TF is a transcriptional activator, and functions as a tumor suppressor. Multiple transcript variants encoding different isoforms have been found for this gene, some of which are implicated in carcinogenesis [45]. TFE3_f1 will bind to TFE3. This TF promotes the expression of genes downstream of transforming growth factor beta (TGF-beta) signaling. This gene may be involved in chromosomal translocations in renal cell carcinomas and other cancers, resulting in the production of usion proteins [46].

TF annotations for top five enriched motifs in all six cell lines are reported in Additional file 1: Tables S8 and S9. From these results, we conclude that sequence embedding features not only perform well but also are interpretable through motif enrichment analysis. Although deep learning is widely applied and always surpass conventional methods in various tasks, it is hard to interpret why deep models perform well. We make use of attention mechanism and try to find out why sequence embedding features outperform others methods. One reasonable explanation is that EP2vec can capture important motifs in a sequence that reveal sequence information.

### Combination of two types of features

According to Table 2, we observe that our sequence embedding features outperform experimental features in TargetFinder and sequence features computed in gkmSVM and SPEID. Here, to further improve the prediction accuracy of our model, we attempt to combine our sequence embedding features and experimental features in TargetFinder.

**Fig. 4:**
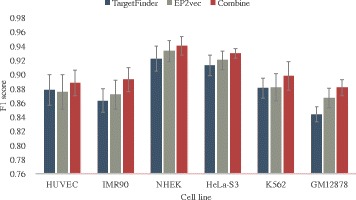
The F1 scores of combined features and two single types of features in 10-fold cross-validation. The combination of both types of features generate even better performance, indicating sequence embedding features and experiment features can be complementary to each other

Concretely, we concatenate the 200-dimensional sequence embedding features and the experimental features and then we use a GBRT with the same hyper-parameters as EP2vec to train a classifier for predicting EPIs. According to Fig. 4, we can see that sequence embedding features are better than experimental features in capturing useful sequence information, while the combination of both types of features generate even better performance. Consequently, sequence embedding features in enhancers and promoters and experimental features in windows between enhancers and promoters facilitate each other and combination of them performs better than all other feature sets. Finally, we conclude that sequence embedding features and experiment features can be complementary to each other and we could take advantage of existing experimental features and extracted sequence embedding features to predict true EPIs with high accuracy.

## Discussion

Deep learning has successful applications in both computer vision and natural language processing (NLP). As is well known, Convolutional Neural Network (CNN) is a powerful deep learning model in computer vision area. Inspired by deep learning applied in image processing, DeepSEA and DeepBind first regard DNA sequences as binary images through one-hot encoding. They both preprocess the DNA sequences by transforming them into 4xL images (L is the length of a sequence), and then use a CNN to model DNA sequences. Many other deep learning approaches applied in sequence analysis recently all follow this idea and achieve excellent performance.

Our deep learning framework EP2vec is different from them. We solve this sequence analysis problem from a different perspective inspired from NLP. In fact, there are also many successful applications of deep learning in NLP area, such as word2vec which embeds words into a vector space. Paragraph Vector is based on word2vec, and it embeds whole sentences to vectors encoding their semantic meanings. We think it is more natural to treat a DNA sequence as a sentence other than an image, since the DNA sequence is only a one-dimensional data while images are often two-dimensional data. Hence, we regard a DNA sequence as a sentence which is comprised of *k*-mers (or words). We learn good representations of DNA sequences using Paragraph Vector as the results shown in Computational performance. In this unsupervised feature extraction stage, we apply deep learning to extract sequence embedding features which will be used in supervised classification. The superiority of our framework mainly lies in that we utilize the global statistics of *k*-mer relationships, and can learn a global representation of a DNA sequence.

Our method is innovative in using a different deep learning diagram from existing methods in the following several aspects:

First, we draw strength from recent advance in deep learning and successfully extract fixed-length embedding features for variable-length sequences. Our results suggest that it is possible to use only sequence embedding features instead of traditional genomic and epigenomic features to predict EPIs with competitive results, and that DNA sequences themselves provide enough information about what function they perform in different cell lines. Different from other computational features for DNA sequences, we learn the sequence embedding features on basis of the *k*-mer co-occurrence statistics using Paragraph Vector, and by learning an embedding vector directly for a sequence we can better represent the global sequence information.

Second, we carry out sensitivity analysis with regard to model hyper-parameters involved in the unsupervised feature learning stage. The result indicates that EP2vec is robust to its hyper-parameters and is effective in capturing the information of whole sequences. Even using only 10-dimensional sequence embedding features, EP2vec still yields satisfactory results.

Third, we explore important motifs that account for enhancers and promoter when mining the information in sequence embedding features. As we all know, deep learning often behaves like black box and people find it hard to explain what the extracted features mean. We illustrate the meaning of sequence embedding features by visualizing the motifs found by attention mechanism with sequence logo. These results indicate that sequence embedding features have underlying biological meanings which we need to pay more attention to.

Last but not the least, we train a hybrid model using both sequence embedding features and the experimental features, which generates better classification results than using a single type of features. We conclude that the two types of features are complementary to each other, and their combination is beneficial for prediction of EPIs.

Nevertheless, our approach can still be improved in the following several aspects. First, we treat every word equally without discrimination in the training. Nevertheless, using the attention mechanism, we pay more attention on important words in the visualizing process. Hence, we could adopt attention mechanism in the training process and gain better representation of the whole sequence. Second, in the unsupervised feature extraction stage of EP2vec workflow, we train sequence embedding features for enhancers and promoters separately, without using interaction information. In fact, we can inject the EPIs label information in this stage, so that we can encode not only the cell line specific information of enhancer and promoter sequences but also the paired information of enhancers and promoters in the feature representation. Third, we could combine sequence-based features and massive biological experiments data in the network training process for sequence embedding features. Although sequence features show good performance, they lose cell line specific information which is enriched in experimental features. We can fuse the cell line specific experimental features in training process and predict EPIs genome-wide.

## Conclusions

In conclusion, EP2vec extracts sequence embedding features using unsupervised deep learning method and predicts EPIs accurately using GBRT classifier achieving state-of-the-art performance. Different from the previous sequence-based methods, EP2vec is innovative in extracting fixed-length embedding features for variable-length sequences and retaining the context information. Given the excellent performance of EP2vec, we will continue to improve our approach according to the above discussion. We expect EP2vec and the future revised version to play an important role in all kinds of sequence prediction tasks, such as identification of miRNA target sites and RNA-RNA interactions, and benefit further downstream analysis.

## Additional file

Additional file 1: Supplementary Tables and Supplementary Figures. (DOCX 302 kb)

## Abbreviations

1D: One-dimensional
3C: Chromosome conformation capture-based
3D: Three-dimensional
auPRC: area under the Precision Recall curve
auROC: area under the Receiver Operating Characteristic curve
CART: Classification and regression trees
ChIA-PET: Chromatin interaction analysis by paired-end tag sequencing
CNN: Convolutional neural networks
EPI: Enhancer-promoter interactions
GBRT: Gradient boosted regression trees
GWAS: Genome-wide association studies
lncRNA: long noncoding RNA
LSTM: Long short-term memory
NLP: Natural language processing
SGD: Stochastic gradient descent
SNP: Single nucleotide polymorphism
