# Supplementary material for: Prediction of enhancer-promoter interactions via natural language processing
Source: BMC Genomics. 2018 May 9;19(Suppl 2):84. doi: 10.1186/s12864-018-4459-6 (PMC5954283; doi:10.1186/s12864-018-4459-6)
Supplement: Supplementary file 1 — Supplementary Tables and Supplementary Figures. (DOCX 302 kb) [file 12864_2018_4459_MOESM1_ESM.docx]

**Supplementary Table 1. The mean values and standard deviations of auROC scores for EP2vec, TargetFinder, Combined, gkmSVM, and SPEID in 10-fold cross-validation experiments.** Combined indicates the method using combination of sequence embedding features and experimental features as described in Section “Results”.

| Dataset | EP2vec | TargetFinder | Combined | gkmSVM | SPEID |
| --- | --- | --- | --- | --- | --- |
| K562 | 0.912(0.019) | 0.950(0.013) | 0.953(0.012) | 0.876(0.016) | 0.904(0.021) |
| IMR90 | 0.901(0.017) | 0.940(0.015) | 0.948(0.011) | 0.828(0.028) | 0.880(0.029) |
| GM12878 | 0.902(0.017) | 0.924(0.010) | 0.943(0.008) | 0.840(0.008) | 0.875(0.015) |
| HUVEC | 0.900(0.025) | 0.948(0.012) | 0.943(0.013) | 0.819(0.019) | 0.874(0.020) |
| HeLa-S3 | 0.937(0.015) | 0.971(0.006) | 0.970(0.009) | 0.889(0.015) | 0.927(0.022) |
| NHEK | 0.954(0.012) | 0.974(0.009) | 0.976(0.010) | 0.881(0.022) | 0.943(0.014) |
| FANTOM | 0.907(0.016) | / | / | 0.843(0.024) | / |

**Supplementary Table 2. The mean values and standard deviations of auPRC scores for EP2vec, TargetFinder, Combined, gkmSVM, and SPEID in 10-fold cross-validation experiments.** Combined indicates the method using combination of sequence embedding features and experimental features as described in Section “Results”.

| Dataset | EP2vec | TargetFinder | Combined | gkmSVM | SPEID |
| --- | --- | --- | --- | --- | --- |
| K562 | 0.915(0.021) | 0.962(0.008) | 0.964(0.009) | 0.876(0.023) | 0.909(0.023) |
| IMR90 | 0.905(0.020) | 0.952(0.011) | 0.953(0.011) | 0.842(0.037) | 0.883(0.028) |
| GM12878 | 0.909(0.019) | 0.937(0.007) | 0.948(0.011) | 0.847(0.015) | 0.889(0.014) |
| HUVEC | 0.897(0.036) | 0.957(0.010) | 0.949(0.014) | 0.827(0.023) | 0.878(0.014) |
| HeLa-S3 | 0.935(0.013) | 0.976(0.005) | 0.976(0.008) | 0.892(0.026) | 0.931(0.019) |
| NHEK | 0.946(0.022) | 0.980(0.006) | 0.981(0.007) | 0.889(0.027) | 0.931(0.021) |
| FANTOM | 0.906(0.015) | / | / | 0.833(0.033) | / |

**Supplementary Table 3. F1 scores with different parameters, including *k*, stride *s* and embedding vector dimension *d*.** Each column represents one cell line and each row represents different combination of hyper-parameters *k*, stride *s* and embedding dimension *d*. Each cell is the F1 score of EP2vec in this cell with this combination of hyper-parameters. We find that there is no dramatic difference in the same cell line, indicating sequence embedding features capture the fundamental information for the whole sequences no matter how we split the sequences. Note that, even when the dimension *d* drops to only 10, the performance decreases but is still high.

| parameters | K562 | IMR90 | GM12878 | HUVEC | HeLa-S3 | NHEK |
| --- | --- | --- | --- | --- | --- | --- |
| *k* = 6, *s* = 1, *d* = 100 | 0.882(0.019) | 0.872(0.020) | 0.867(0.014) | 0.875(0.024) | 0.920(0.013) | 0.933(0.015) |
| *k* = 7, *s* = 1, *d* = 100 | 0.884(0.018) | 0.867(0.018) | 0.865(0.012) | 0.866(0.019) | 0.920(0.012) | 0.933(0.015) |
| *k* = 8, *s* = 1, *d* = 100 | 0.879(0.021) | 0.872(0.017) | 0.868(0.013) | 0.873(0.025) | 0.919(0.012) | 0.932(0.015) |
| *k* = 9, *s* = 1, *d* = 100 | 0.880(0.020) | 0.868(0.018) | 0.866(0.011) | 0.862(0.021) | 0.921(0.013) | 0.933(0.015) |
| *k* = 10, *s* = 1, *d* = 100 | 0.883(0.021) | 0.869(0.021) | 0.868(0.012) | 0.860(0.021) | 0.921(0.012) | 0.930(0.014) |
| *k* = 6, *s* = 1, *d* = 100 | 0.882(0.019) | 0.872(0.020) | 0.867(0.014) | 0.875(0.024) | 0.920(0.013) | 0.933(0.015) |
| *k* = 6, *s* = 2, *d* = 100 | 0.882(0.021) | 0.869(0.018) | 0.868(0.009) | 0.872(0.025) | 0.921(0.013) | 0.932(0.015) |
| *k* = 6, *s* = 3, *d* = 100 | 0.881(0.020) | 0.868(0.018) | 0.865(0.015) | 0.871(0.019) | 0.921(0.013) | 0.933(0.015) |
| *k* = 6, *s* = 4, *d* = 100 | 0.883(0.022) | 0.866(0.019) | 0.867(0.013) | 0.875(0.020) | 0.921(0.012) | 0.932(0.016) |
| *k* = 6, *s* = 5, *d* = 100 | 0.881(0.022) | 0.865(0.018) | 0.864(0.012) | 0.871(0.022) | 0.921(0.013) | 0.934(0.015) |
| *k* = 6, *s* = 6, *d* = 100 | 0.883(0.021) | 0.866(0.017) | 0.866(0.014) | 0.870(0.022) | 0.921(0.012) | 0.932(0.015) |
| *k* = 6, *s* = 1, *d* = 10 | 0.836(0.028) | 0.785(0.031) | 0.796(0.019) | 0.800(0.025) | 0.862(0.015) | 0.890(0.012) |
| *k* = 6, *s* = 1, *d* = 20 | 0.860(0.019) | 0.842(0.028) | 0.84(0.014) | 0.826(0.021) | 0.904(0.012) | 0.917(0.013) |
| *k* = 6, *s* = 1, *d* = 40 | 0.878(0.021) | 0.868(0.020) | 0.855(0.015) | 0.850(0.025) | 0.914(0.012) | 0.931(0.016) |
| *k* = 6, *s* = 1, *d* = 80 | 0.880(0.021) | 0.869(0.019) | 0.865(0.015) | 0.870(0.024) | 0.920(0.014) | 0.933(0.015) |
| *k* = 6, *s* = 1, *d* = 100 | 0.882(0.019) | 0.872(0.020) | 0.867(0.014) | 0.875(0.024) | 0.920(0.013) | 0.933(0.015) |
| *k* = 6, *s* = 1, *d* = 200 | 0.882(0.021) | 0.869(0.019) | 0.870(0.014) | 0.878(0.024) | 0.921(0.012) | 0.933(0.016) |
| *k* = 6, *s* = 1, *d* = 400 | 0.882(0.021) | 0.868(0.019) | 0.868(0.014) | 0.878(0.025) | 0.921(0.012) | 0.933(0.015) |

**Supplementary Table 4. auROC scores with different parameters, including *k*, stride *s* and embedding vector dimension *d*.** Each column represents a cell line and each row represents different combination of hyper-parameters *k*, stride *s* and embedding dimension *d*. Each cell is the AUC score of EP2vec in this cell with this combination of hyper-parameters. We find that there is no dramatic difference in the same cell line, indicating sequence embedding features capture the fundamental information for the whole sequences no matter how we split the sequences. Note that, even when the dimension *d* drops to only 10, the performance decreases but is still high.

| parameters | K562 | IMR90 | GM12878 | HUVEC | HeLa-S3 | NHEK |
| --- | --- | --- | --- | --- | --- | --- |
| *k* = 6, *s* = 1, *d* = 100 | 0.912(0.019) | 0.901(0.017) | 0.902(0.017) | 0.900(0.025) | 0.937(0.015) | 0.954(0.012) |
| *k* = 7, *s* = 1, *d* = 100 | 0.908(0.016) | 0.903(0.015) | 0.903(0.016) | 0.896(0.025) | 0.933(0.013) | 0.950(0.013) |
| *k* = 8, *s* = 1, *d* = 100 | 0.913(0.019) | 0.899(0.016) | 0.903(0.017) | 0.900(0.024) | 0.935(0.013) | 0.949(0.017) |
| *k* = 9, *s* = 1, *d* = 100 | 0.909(0.016) | 0.900(0.017) | 0.900(0.018) | 0.900(0.023) | 0.938(0.012) | 0.950(0.015) |
| *k* = 10, *s* = 1, *d* = 100 | 0.915(0.018) | 0.902(0.017) | 0.901(0.016) | 0.898(0.021) | 0.938(0.014) | 0.952(0.014) |
| *k* = 6, *s* = 1, *d* = 100 | 0.912(0.019) | 0.901(0.017) | 0.902(0.017) | 0.900(0.025) | 0.937(0.015) | 0.954(0.012) |
| *k* = 6, *s* = 2, *d* = 100 | 0.911(0.018) | 0.898(0.014) | 0.900(0.017) | 0.898(0.027) | 0.937(0.014) | 0.953(0.015) |
| *k* = 6, *s* = 3, *d* = 100 | 0.912(0.015) | 0.900(0.019) | 0.902(0.015) | 0.899(0.023) | 0.940(0.011) | 0.949(0.011) |
| *k* = 6, *s* = 4, *d* = 100 | 0.914(0.018) | 0.899(0.019) | 0.901(0.017) | 0.906(0.023) | 0.940(0.010) | 0.955(0.014) |
| *k* = 6, *s* = 5, *d* = 100 | 0.911(0.019) | 0.898(0.016) | 0.904(0.017) | 0.905(0.022) | 0.939(0.011) | 0.952(0.018) |
| *k* = 6, *s* = 6, *d* = 100 | 0.914(0.017) | 0.900(0.013) | 0.901(0.015) | 0.903(0.022) | 0.938(0.012) | 0.949(0.012) |
| *k* = 6, *s* = 1, *d* = 10 | 0.893(0.025) | 0.858(0.029) | 0.860(0.023) | 0.862(0.022) | 0.917(0.014) | 0.934(0.013) |
| *k* = 6, *s* = 1, *d* = 20 | 0.901(0.020) | 0.890(0.021) | 0.888(0.018) | 0.883(0.020) | 0.932(0.012) | 0.948(0.013) |
| *k* = 6, *s* = 1, *d* = 40 | 0.910(0.016) | 0.898(0.018) | 0.898(0.017) | 0.895(0.026) | 0.935(0.012) | 0.952(0.014) |
| *k* = 6, *s* = 1, *d* = 80 | 0.909(0.019) | 0.902(0.017) | 0.899(0.019) | 0.901(0.022) | 0.937(0.014) | 0.956(0.014) |
| *k* = 6, *s* = 1, *d* = 100 | 0.912(0.019) | 0.901(0.017) | 0.902(0.017) | 0.900(0.025) | 0.937(0.015) | 0.954(0.012) |
| *k* = 6, *s* = 1, *d* = 200 | 0.915(0.018) | 0.904(0.014) | 0.901(0.018) | 0.901(0.024) | 0.938(0.015) | 0.952(0.015) |
| *k* = 6, *s* = 1, *d* = 400 | 0.910(0.018) | 0.905(0.016) | 0.901(0.016) | 0.905(0.025) | 0.937(0.013) | 0.954(0.013) |

**Supplementary Table 5. auPRC scores with different parameters, including *k*, stride *s* and embedding vector dimension *d*.** Each column represents a cell line and each row represents different combination of hyper-parameters *k*, stride *s* and embedding dimension *d*. Each cell is the AUC score of EP2vec in this cell with this combination of hyper-parameters. We find that there is no dramatic difference in the same cell line, indicating sequence embedding features capture the fundamental information for the whole sequences no matter how we split the sequences. Note that, even when the dimension *d* drops to only 10, the performance decreases but is still high.

| parameters | K562 | IMR90 | GM12878 | HUVEC | HeLa-S3 | NHEK |
| --- | --- | --- | --- | --- | --- | --- |
| *k* = 6, *s* = 1, *d* = 100 | 0.915(0.021) | 0.905(0.020) | 0.909(0.019) | 0.897(0.036) | 0.935(0.013) | 0.946(0.022) |
| *k* = 7, *s* = 1, *d* = 100 | 0.913(0.019) | 0.909(0.015) | 0.908(0.015) | 0.898(0.033) | 0.931(0.013) | 0.946(0.022) |
| *k* = 8, *s* = 1, *d* = 100 | 0.916(0.022) | 0.904(0.019) | 0.909(0.016) | 0.902(0.032) | 0.934(0.012) | 0.946(0.026) |
| *k* = 9, *s* = 1, *d* = 100 | 0.915(0.019) | 0.906(0.019) | 0.907(0.017) | 0.899(0.034) | 0.936(0.011) | 0.947(0.020) |
| *k* = 10, *s* = 1, *d* = 100 | 0.918(0.022) | 0.906(0.023) | 0.907(0.015) | 0.899(0.035) | 0.936(0.013) | 0.948(0.021) |
| *k* = 6, *s* = 1, *d* = 100 | 0.915(0.021) | 0.905(0.020) | 0.909(0.019) | 0.897(0.036) | 0.935(0.013) | 0.946(0.022) |
| *k* = 6, *s* = 2, *d* = 100 | 0.913(0.024) | 0.904(0.023) | 0.906(0.016) | 0.900(0.035) | 0.937(0.015) | 0.946(0.025) |
| *k* = 6, *s* = 3, *d* = 100 | 0.913(0.019) | 0.905(0.023) | 0.910(0.017) | 0.902(0.035) | 0.939(0.011) | 0.946(0.018) |
| *k* = 6, *s* = 4, *d* = 100 | 0.916(0.021) | 0.904(0.022) | 0.907(0.017) | 0.909(0.031) | 0.943(0.009) | 0.951(0.018) |
| *k* = 6, *s* = 5, *d* = 100 | 0.913(0.023) | 0.902(0.020) | 0.907(0.018) | 0.908(0.031) | 0.942(0.013) | 0.949(0.022) |
| *k* = 6, *s* = 6, *d* = 100 | 0.914(0.023) | 0.904(0.017) | 0.905(0.016) | 0.906(0.034) | 0.943(0.009) | 0.948(0.017) |
| *k* = 6, *s* = 1, *d* = 10 | 0.895(0.028) | 0.866(0.024) | 0.862(0.026) | 0.856(0.030) | 0.918(0.008) | 0.928(0.027) |
| *k* = 6, *s* = 1, *d* = 20 | 0.906(0.025) | 0.893(0.025) | 0.893(0.020) | 0.879(0.030) | 0.931(0.012) | 0.943(0.022) |
| *k* = 6, *s* = 1, *d* = 40 | 0.912(0.018) | 0.901(0.025) | 0.903(0.017) | 0.895(0.037) | 0.933(0.010) | 0.945(0.023) |
| *k* = 6, *s* = 1, *d* = 80 | 0.912(0.024) | 0.906(0.020) | 0.907(0.017) | 0.903(0.029) | 0.936(0.014) | 0.952(0.020) |
| *k* = 6, *s* = 1, *d* = 100 | 0.915(0.021) | 0.905(0.020) | 0.909(0.019) | 0.897(0.036) | 0.935(0.013) | 0.946(0.022) |
| *k* = 6, *s* = 1, *d* = 200 | 0.916(0.022) | 0.910(0.019) | 0.909(0.016) | 0.900(0.033) | 0.935(0.011) | 0.941(0.025) |
| *k* = 6, *s* = 1, *d* = 400 | 0.911(0.022) | 0.908(0.024) | 0.907(0.016) | 0.906(0.032) | 0.937(0.012) | 0.953(0.017) |

**Supplementary Table 6.** **The motif enrichment analysis results of CentriMo for enhancers**. We pick out the most informative *k*-mer in every enhancer in positive samples and use these sequences to search for enriched motifs. We select HOCOMOCO (v9) database to test for enrichment and present all the enrichment results in the following table. Note that, different cell lines might have different numbers of enriched motifs.

| Cell lines | Motif ID | Motif Name | *E*-value |
| --- | --- | --- | --- |
| K562 | KLF6_si | KLF6 | 2.6e-19 |
|  | NKX25_f1 | NKX25 | 2.3e-8 |
|  | TFE3_f1 | TFE3 | 9.6e-8 |
|  | AP2B_f1 | AP2B | 1.8e-5 |
|  | ETV4_f1 | ETV4 | 2.3e-3 |
|  | NR1I2_si | NR1I2 | 3.8e-3 |
|  | RUNX1_f1 | RUNX1 | 1.1e-2 |
|  | TGIF1_si | TGIF1 | 1.3e-2 |
|  | HXD10_f1 | HXD10 | 2.3e-2 |
|  | MAFG_si | MAFG | 7.2e-2 |
|  | MAF_f1 | MAF | 7.2e-2 |
|  | CRX_si | CRX | 8.7e-2 |
|  | NOBOX_si | NOBOX | 2.1e-1 |
|  | USF1_f1 | USF1 | 2.3e-1 |
|  | FOXJ2_f1 | FOXJ2 | 3.7e-1 |
|  | PRRX1_f1 | PRRX1 | 3.9e-1 |
|  | CEBPB_f1 | CEBPB | 4.1e-1 |
|  | MYBB_f1 | MYBB | 8.8e-1 |
|  | FOXJ2_f1 | FOXJ2 | 3.7e-1 |
|  | PRRX1_f1 | PRRX1 | 3.9e-1 |
| IMR90 | SOX5_f1 | SOX5 | 1.2e-4 |
|  | AP2C_f1 | AP2C | 1.0e-3 |
|  | E2F3_si | E2F3 | 1.3e-2 |
|  | ERR2_f1 | ERR2 | 1.6e-2 |
|  | AHR_si | AHR | 8.6e-2 |
|  | NFAC2_f1 | NFAC2 | 1.3e-1 |
|  | NFIA+NFIB+NFIC+NFIX_si | NFIA+NFIB+NFIC+NFIX | 5.2e-1 |
|  | FOXO4_f1 | FOXO4 | 9.7e-1 |
| GM12878 | OTX1_f1 | OTX1 | 2.0e-8 |
|  | CRX_si | CRX | 1.1e-7 |
|  | NKX21_f1 | NKX21 | 2.3e-6 |
|  | GATA6_f2 | GATA6 | 1.7e-5 |
|  | ZIC2_f1 | ZIC2 | 4.7e-5 |
|  | NFAC3_f1 | NFAC3 | 1.3e-3 |
|  | RXRB_f1 | RXRB | 2.1e-3 |
|  | HMGA1_f1 | HMGA1 | 3.0e-2 |
|  | AP2C_f1 | AP2C | 1.2e-1 |
|  | UBIP1_f1 | UBIP1 | 1.2e-1 |
|  | ETS1_si | ETS1 | 1.3e-1 |
|  | NOBOX_si | NOBOX | 1.9e-1 |
|  | FOXD3_f1 | FOXD3 | 2.2e-1 |
|  | THB_f1 | THB | 3.3e-1 |
|  | CEBPB_f1 | CEBPB | 4.1e-1 |
|  | ELK1_f1 | ELK1 | 6.8e-1 |
|  | SMAD3_f1 | SMAD3 | 9.0e-1 |
|  | JUN_f1 | JUN | 9.7e-1 |
| HUVEC | MYB_f1 | MYB | 2.0e-5 |
|  | GFI1_f1 | GFI1 | 1.8e-4 |
|  | KLF6_si | KLF6 | 2.0e-3 |
|  | IKZF1_f1 | IKZF1 | 9.1e-3 |
|  | OVOL1_f1 | OVOL1 | 1.9e-2 |
|  | THA_f1 | THA | 6.2e-2 |
|  | RUNX1_f1 | RUNX1 | 7.1e-2 |
|  | DLX3_do | DLX3 | 1.9e-1 |
|  | JUN_f1 | JUN | 3.0e-1 |
| HeLa-S3 | GATA6_f2 | GATA6 | 6.5e-28 |
|  | OTX1_f1 | OTX1 | 6.9e-11 |
|  | CDX2_f1 | CDX2 | 5.6e-10 |
|  | CRX_si | CRX | 2.1e-9 |
|  | JUNB_f1 | JUNB | 1.0e-8 |
|  | PRRX2_f1 | PRRX2 | 3.8e-7 |
|  | NR4A2_si | NR4A2 | 4.0e-7 |
|  | FOSB_f1 | FOSB | 2.0e-5 |
|  | HXD4_f1 | HXD4 | 5.1e-4 |
|  | TWST1_f1 | TWST1 | 9.2e-4 |
|  | NFAC3_f1 | NFAC3 | 9.5e-4 |
|  | NR4A1_f1 | NR4A1 | 3.3e-3 |
|  | NF2L1_f1 | NF2L1 | 6.1e-3 |
|  | MAF_f1 | MAF | 8.9e-3 |
|  | KLF6_si | KLF6 | 1.4e-2 |
|  | GATA3_si | GATA3 | 1.7e-2 |
|  | RARG_f1 | RARG | 1.7e-2 |
|  | TGIF1_si | TGIF1 | 3.8e-2 |
|  | PPARA_f1 | PPARA | 3.8e-2 |
|  | ERR2_f1 | ERR2 | 8.3e-2 |
|  | ESR2_si | ESR2 | 1.1e-1 |
|  | NKX25_f1 | NKX25 | 3.0e-1 |
|  | FOXD3_f1 | FOXD3 | 3.5e-1 |
|  | NR0B1_si | NR0B1 | 5.1e-1 |
|  | JUN_f1 | JUN | 5.5e-1 |
|  | MYB_f1 | MYB | 6.7e-1 |
|  | UBIP1_f1 | UBIP1 | 7.3e-1 |
|  | ISL1_f1 | ISL1 | 7.5e-1 |
|  | GATA2_si | GATA2 | 8.9e-1 |
| NHEK | TFE3_f1 | TFE3 | 4.1e-8 |
|  | NF2L1_f1 | NF2L1 | 5.8e-7 |
|  | USF1_f1 | USF1 | 3.3e-6 |
|  | SNAI1_f1 | SNAI1 | 5.3e-6 |
|  | NKX25_f1 | NKX25 | 7.0e-5 |
|  | LEF1_f1 | LEF1 | 2.0e-4 |
|  | MAFG_si | MAFG | 2.1e-3 |
|  | MYF6_f1 | MYF6 | 3.0e-3 |
|  | SNAI2_f1 | SNAI2 | 1.0e-2 |
|  | FOXO4_f1 | FOXO4 | 1.3e-2 |
|  | RUNX1_f1 | RUNX1 | 3.3e-1 |
|  | KLF8_f1 | KLF8 | 6.8e-1 |
|  | FOXP2_si | FOXP2 | 8.2e-1 |

**Supplementary Table 7. The motif enrichment analysis results of CentriMo for promoters.** We pick out the most informative *k*-mer in every promoter in positive samples and use these sequences to search for enriched motifs. We select HOCOMOCO (v9) database to test for enrichment and present all the enrichment results in the following table. Note that, different cell lines might have different numbers of enriched motifs.

| Cell lines | Motif ID | Motif Name | *E*-value |
| --- | --- | --- | --- |
| K562 | PPARA_f1 | PPARA | 4.0e-11 |
|  | MAF_f1 | MAF | 8.2e-10 |
|  | THA_f1 | THA | 9.4e-9 |
|  | GCR_si | GCR | 7.3e-8 |
|  | COT2_f1 | COT2 | 1.8e-7 |
|  | NKX25_f1 | NKX25 | 9.0e-6 |
|  | RARG_f1 | RARG | 5.7e-5 |
|  | NF2L1_f1 | NF2L1 | 2.4e-4 |
|  | KLF8_f1 | KLF8 | 2.2e-3 |
|  | KLF6_si | KLF6 | 2.3e-3 |
|  | NR4A2_si | NR4A2 | 2.6e-2 |
|  | UBIP1_f1 | UBIP1 | 7.7e-2 |
|  | NKX28_f1 | NKX28 | 1.0e-1 |
|  | ISL1_f1 | ISL1 | 3.9e-1 |
|  | COT1_si | COT1 | 5.7e-1 |
|  | HBP1_f1 | HBP1 | 7.3e-1 |
| IMR90 | LEF1_f1 | LEF1 | 1.1e-8 |
|  | SOX10_si | SOX10 | 3.3e-8 |
|  | MSX2_f1 | MSX2 | 5.8e-4 |
|  | KLF4_f2 | KLF4 | 3.5e-3 |
|  | SMAD3_f1 | SMAD3 | 4.1e-3 |
|  | GATA4_f1 | GATA4 | 1.3e-2 |
|  | JUN_f1 | JUN | 5.9e-2 |
|  | SOX15_f1 | SOX15 | 7.5e-2 |
|  | PITX2_si | PITX2 | 7.8e-2 |
|  | VDR_f1 | VDR | 1.7e-1 |
|  | OVOL1_f1 | OVOL1 | 3.2e-1 |
|  | CREB1_f1 | CREB1 | 8.3e-1 |
| GM12878 | CXXC1_si | CXXC1 | 2.0e-39 |
|  | SOX15_f1 | SOX15 | 3.8e-31 |
|  | PRRX1_f1 | PRRX1 | 2.7e-14 |
|  | THB_f1 | THB | 2.2e-11 |
|  | MSX2_f1 | MSX2 | 6.3e-10 |
|  | PDX1_do | PDX1 | 1.2e-6 |
|  | DLX3_do | DLX3 | 4.1e-6 |
|  | COT1_si | COT1 | 2.6e-5 |
|  | DLX2_f1 | DLX2 | 4.4e-5 |
|  | GABP1+GABP2_f1 | GABP1+GABP2 | 1.4e-4 |
|  | NKX25_f1 | NKX25 | 1.6e-4 |
|  | HMGA1_f1 | HMGA1 | 5.3e-4 |
|  | CRX_si | CRX | 1.1e-3 |
|  | PPARG_si | PPARG | 1.2e-3 |
|  | PRRX2_f1 | PRRX2 | 5.7e-3 |
|  | ETV4_f1 | ETV4 | 1.6e-2 |
|  | BATF_si | BATF | 1.9e-1 |
|  | NR5A2_f1 | NR5A2 | 5.8e-1 |
| HUVEC | MYB_f1 | MYB | 5.3e-4 |
|  | SOX15_f1 | SOX15 | 2.1e-3 |
|  | CXXC1_si | CXXC1 | 4.0e-3 |
|  | GABP1+GABP2_f1 | GABP1+GABP2 | 4.3e-3 |
|  | NKX21_f1 | NKX21 | 5.6e-3 |
|  | PAX2_si | PAX2 | 2.2e-2 |
|  | SMRC1_f1 | SMRC1 | 2.1e-1 |
|  | NR5A2_f1 | NR5A2 | 5.8e-1 |
|  | HBP1_f1 | HBP1 | 7.3e-1 |
|  | ZN333_f1 | ZN333 | 7.3e-1 |
|  | MSX2_f1 | MSX2 | 7.6e-1 |
| HeLa-S3 | DLX2_f1 | DLX2 | 1.7e-7 |
|  | DLX3_do | DLX3 | 3.7e-7 |
|  | PRGR_f1 | PRGR | 9.5e-4 |
|  | PRRX2_f1 | PRRX2 | 1.4e-3 |
|  | HXB7_si | HXB7 | 2.4e-3 |
|  | NR1I3_si | NR1I3 | 2.8e-3 |
|  | NR1I2_si | NR1I2 | 7.8e-3 |
|  | MYB_f1 | MYB | 3.4e-2 |
|  | RXRB_f1 | RXRB | 8.2e-2 |
|  | SOX15_f1 | SOX15 | 1.8e-1 |
|  | ETV4_f1 | ETV4 | 3.0e-1 |
|  | JUN_f1 | JUN | 3.9e-1 |
|  | PAX5_si | PAX5 | 3.9e-1 |
|  | FOXP2_si | FOXP2 | 4.6e-1 |
|  | MYCN_si | MYCN | 6.7e-1 |
|  | RARG_f1 | RARG | 6.7e-1 |
|  | RUNX1_f1 | RUNX1 | 7.0e-1 |
|  | BHE40_f2 | BHE40 | 8.3e-1 |
|  | ERR3_f1 | ERR3 | 8.3e-1 |
| NHEK | KLF6_si | KLF6 | 9.3e-2 |
|  | PAX2_si | PAX2 | 3.9e-1 |
|  | MAFB_f1 | MAFB | 5.9e-1 |
|  | LEF1_f1 | LEF1 | 6.6e-1 |
|  | SOX15_f1 | SOX15 | 9.5e-1 |

**Supplementary Table 8. The TF annotations for top five enriched motifs in enhancers for six different cell lines.** Although motif enrichment analysis is simple but the enriched motifs do represent some cell line-specific information, for example, enriched motifs in cancer-related cell lines might play a role in carcinogenesis.

| Cell lines | TF name | TF annotation |
| --- | --- | --- |
| K562 | KLF6 | This gene encodes a member of the Kruppel-like family of transcription factors. The zinc finger protein is a transcriptional activator, and functions as a tumor suppressor. Multiple transcript variants encoding different isoforms have been found for this gene, some of which are implicated in carcinogenesis. [provided by RefSeq, May 2009] |
|  | NKX25 | This gene encodes a homeobox-containing transcription factor. This transcription factor functions in heart formation and development. Mutations in this gene cause atrial septal defect with atrioventricular conduction defect, and also tetralogy of Fallot, which are both heart malformation diseases. Mutations in this gene can also cause congenital hypothyroidism non-goitrous type 5, a non-autoimmune condition. Alternative splicing results in multiple transcript variants. [provided by RefSeq, Oct 2009] |
|  | TFE3 | This gene encodes a basic helix-loop-helix domain-containing transcription factor that binds MUE3-type E-box sequences in the promoter of genes. The encoded protein promotes the expression of genes downstream of transforming growth factor beta (TGF-beta) signaling. This gene may be involved in chromosomal translocations in renal cell carcinomas and other cancers, resulting in the production of fusion proteins. Translocation partners include PRCC (papillary renal cell carcinoma), NONO (non-POU domain containing, octamer-binding), and ASPSCR1 (alveolar soft part sarcoma chromosome region, candidate 1), among other genes. Alternative splicing results in multiple transcript variants. [provided by RefSeq, Aug 2013] |
|  | AP2B | This gene encodes a member of the AP-2 family of transcription factors. AP-2 proteins form homo- or hetero-dimers with other AP-2 family members and bind specific DNA sequences. They are thought to stimulate cell proliferation and suppress terminal differentiation of specific cell lines during embryonic development. Specific AP-2 family members differ in their expression patterns and binding affinity for different promoters. This protein functions as both a transcriptional activator and repressor. Mutations in this gene result in autosomal dominant Char syndrome, suggesting that this gene functions in the differentiation of neural crest cell derivatives. [provided by RefSeq, Jul 2008] |
|  | ETV4 | This gene product belongs to the nuclear receptor superfamily, members of which are transcription factors characterized by a ligand-binding domain and a DNA-binding domain. The encoded protein is a transcriptional regulator of the cytochrome P450 gene CYP3A4, binding to the response element of the CYP3A4 promoter as a heterodimer with the 9-cis retinoic acid receptor RXR. It is activated by a range of compounds that induce CYP3A4, including dexamethasone and rifampicin. Several alternatively spliced transcripts encoding different isoforms, some of which use non-AUG (CUG) translation initiation codon, have been described for this gene. Additional transcript variants exist, however, they have not been fully characterized. [provided by RefSeq, Jul 2008] |
| IMR90 | SOX5 | This gene encodes a member of the SOX (SRY-related HMG-box) family of transcription factors involved in the regulation of embryonic development and in the determination of the cell fate. The encoded protein may act as a transcriptional regulator after forming a protein complex with other proteins. The encoded protein may play a role in chondrogenesis. A pseudogene of this gene is located on chromosome 8. Multiple transcript variants encoding distinct isoforms have been identified for this gene. [provided by RefSeq, Jul 2008] |
|  | AP2C | The protein encoded by this gene is a sequence-specific DNA-binding transcription factor involved in the activation of several developmental genes. The encoded protein can act as either a homodimer or heterodimer with other family members and is induced during retinoic acid-mediated differentiation. It plays a role in the development of the eyes, face, body wall, limbs, and neural tube. [provided by RefSeq, Jul 2008] |
|  | E2F3 | This gene encodes a member of a small family of transcription factors that function through binding of DP interaction partner proteins. The encoded protein recognizes a specific sequence motif in DNA and interacts directly with the retinoblastoma protein (pRB) to regulate the expression of genes involved in the cell cycle. Altered copy number and activity of this gene have been observed in a number of human cancers. There are pseudogenes for this gene on chromosomes 2 and 17. Alternative splicing results in multiple transcript variants. [provided by RefSeq, Mar 2013] |
|  | ERR2 | This gene encodes a protein with similarity to the estrogen receptor. Its function is unknown; however, a similar protein in mouse plays an essential role in placental development. [provided by RefSeq, Jul 2008] |
|  | AHR | The protein encoded by this gene is a ligand-activated helix-loop-helix transcription factor involved in the regulation of biological responses to planar aromatic hydrocarbons. This receptor has been shown to regulate xenobiotic-metabolizing enzymes such as cytochrome P450. Before ligand binding, the encoded protein is sequestered in the cytoplasm; upon ligand binding, this protein moves to the nucleus and stimulates transcription of target genes. [provided by RefSeq, Sep 2015] |
| GM12878 | OTX1 | This gene encodes a member of the bicoid sub-family of homeodomain-containing transcription factors. The encoded protein acts as a transcription factor and may play a role in brain and sensory organ development. A similar protein in mouse is required for proper brain and sensory organ development and can cause epilepsy. Alternative splicing results in multiple transcript variants. [provided by RefSeq, Jan 2015] |
|  | CRX | The protein encoded by this gene is a photoreceptor-specific transcription factor which plays a role in the differentiation of photoreceptor cells. This homeodomain protein is necessary for the maintenance of normal cone and rod function. Mutations in this gene are associated with photoreceptor degeneration, Leber congenital amaurosis type III and the autosomal dominant cone-rod dystrophy 2. Several alternatively spliced transcript variants of this gene have been described, but the full-length nature of some variants has not been determined. [provided by RefSeq, Jul 2008] |
|  | NKX21 | This gene encodes a protein initially identified as a thyroid-specific transcription factor. The encoded protein binds to the thyroglobulin promoter and regulates the expression of thyroid-specific genes but has also been shown to regulate the expression of genes involved in morphogenesis. Mutations and deletions in this gene are associated with benign hereditary chorea, choreoathetosis, congenital hypothyroidism, and neonatal respiratory distress, and may be associated with thyroid cancer. Multiple transcript variants encoding different isoforms have been found for this gene. This gene shares the symbol/alias 'TTF1' with another gene, transcription termination factor 1, which plays a role in ribosomal gene transcription. [provided by RefSeq, Feb 2014] |
|  | GATA6 | This gene is a member of a small family of zinc finger transcription factors that play an important role in the regulation of cellular differentiation and organogenesis during vertebrate development. This gene is expressed during early embryogenesis and localizes to endo- and mesodermally derived cells during later embryogenesis and thereby plays an important role in gut, lung, and heart development. Mutations in this gene are associated with several congenital defects. [provided by RefSeq, Mar 2012] |
|  | ZIC2 | This gene encodes a member of the ZIC family of C2H2-type zinc finger proteins. This protein functions as a transcriptional repressor and may regulate tissue specific expression of dopamine receptor D1. Expansion of an alanine repeat in the C-terminus of the encoded protein and other mutations in this gene cause holoprosencephaly type 5. Holoprosencephaly is the most common structural anomaly of the human brain. A polyhistidine tract polymorphism in this gene may be associated with increased risk of neural tube defects. This gene is closely linked to a gene encoding zinc finger protein of the cerebellum 5, a related family member on chromosome 13. [provided by RefSeq, Jul 2016] |
| HUVEC | MYB | This gene encodes a protein with three HTH DNA-binding domains that functions as a transcription regulator. This protein plays an essential role in the regulation of hematopoiesis. This gene may be aberrently expressed or rearranged or undergo translocation in leukemias and lymphomas, and is considered to be an oncogene. Alternative splicing results in multiple transcript variants. [provided by RefSeq, Jan 2016] |
|  | GFI1 | This gene encodes a nuclear zinc finger protein that functions as a transcriptional repressor. This protein plays a role in diverse developmental contexts, including hematopoiesis and oncogenesis. It functions as part of a complex along with other cofactors to control histone modifications that lead to silencing of the target gene promoters. Mutations in this gene cause autosomal dominant severe congenital neutropenia, and also dominant nonimmune chronic idiopathic neutropenia of adults, which are heterogeneous hematopoietic disorders that cause predispositions to leukemias and infections. Multiple alternatively spliced variants, encoding the same protein, have been identified for this gene. [provided by RefSeq, Jul 2008] |
|  | KLF6 | This gene encodes a member of the Kruppel-like family of transcription factors. The zinc finger protein is a transcriptional activator, and functions as a tumor suppressor. Multiple transcript variants encoding different isoforms have been found for this gene, some of which are implicated in carcinogenesis. [provided by RefSeq, May 2009] |
|  | IKZF1 | This gene encodes a transcription factor that belongs to the family of zinc-finger DNA-binding proteins associated with chromatin remodeling. The expression of this protein is restricted to the fetal and adult hemo-lymphopoietic system, and it functions as a regulator of lymphocyte differentiation. Several alternatively spliced transcript variants encoding different isoforms have been described for this gene. Most isoforms share a common C-terminal domain, which contains two zinc finger motifs that are required for hetero- or homo-dimerization, and for interactions with other proteins. The isoforms, however, differ in the number of N-terminal zinc finger motifs that bind DNA and in nuclear localization signal presence, resulting in members with and without DNA-binding properties. Only a few isoforms contain the requisite three or more N-terminal zinc motifs that confer high affinity binding to a specific core DNA sequence element in the promoters of target genes. The non-DNA-binding isoforms are largely found in the cytoplasm, and are thought to function as dominant-negative factors. Overexpression of some dominant-negative isoforms have been associated with B-cell malignancies, such as acute lymphoblastic leukemia (ALL). [provided by RefSeq, May 2014] |
|  | OVOL1 | This gene encodes a putative zinc finger containing transcription factor that is highly similar to homologous protein in Drosophila and mouse. Based on known functions in these species, this protein is likely involved in hair formation and spermatogenesis in human as well. [provided by RefSeq, Aug 2011] |
| HeLa-S3 | GATA6 | This gene is a member of a small family of zinc finger transcription factors that play an important role in the regulation of cellular differentiation and organogenesis during vertebrate development. This gene is expressed during early embryogenesis and localizes to endo- and mesodermally derived cells during later embryogenesis and thereby plays an important role in gut, lung, and heart development. Mutations in this gene are associated with several congenital defects. [provided by RefSeq, Mar 2012] |
|  | OTX1 | This gene encodes a member of the bicoid sub-family of homeodomain-containing transcription factors. The encoded protein acts as a transcription factor and may play a role in brain and sensory organ development. A similar protein in mouse is required for proper brain and sensory organ development and can cause epilepsy. Alternative splicing results in multiple transcript variants. [provided by RefSeq, Jan 2015] |
|  | CDX2 | This gene is a member of the caudal-related homeobox transcription factor gene family. The encoded protein is a major regulator of intestine-specific genes involved in cell growth an differentiation. This protein also plays a role in early embryonic development of the intestinal tract. Aberrant expression of this gene is associated with intestinal inflammation and tumorigenesis. [provided by RefSeq, Jan 2012] |
|  | CRX | The protein encoded by this gene is a photoreceptor-specific transcription factor which plays a role in the differentiation of photoreceptor cells. This homeodomain protein is necessary for the maintenance of normal cone and rod function. Mutations in this gene are associated with photoreceptor degeneration, Leber congenital amaurosis type III and the autosomal dominant cone-rod dystrophy 2. Several alternatively spliced transcript variants of this gene have been described, but the full-length nature of some variants has not been determined. [provided by RefSeq, Jul 2008] |
|  | JUNB | JUNB (JunB Proto-Oncogene, AP-1 Transcription Factor Subunit) is a Protein Coding gene. Diseases associated with JUNB include Fumarate Hydratase Deficiency and Anaplastic Large Cell Lymphoma. Among its related pathways are MAPK Signaling: Oxidative Stress Pathway and IL27-mediated signaling events. GO annotations related to this gene include transcription factor activity, sequence-specific DNA binding and transcription factor binding. An important paralog of this gene is JUN. |
| NHEK | TFE3 | This gene encodes a basic helix-loop-helix domain-containing transcription factor that binds MUE3-type E-box sequences in the promoter of genes. The encoded protein promotes the expression of genes downstream of transforming growth factor beta (TGF-beta) signaling. This gene may be involved in chromosomal translocations in renal cell carcinomas and other cancers, resulting in the production of fusion proteins. Translocation partners include PRCC (papillary renal cell carcinoma), NONO (non-POU domain containing, octamer-binding), and ASPSCR1 (alveolar soft part sarcoma chromosome region, candidate 1), among other genes. Alternative splicing results in multiple transcript variants. [provided by RefSeq, Aug 2013] |
|  | NF2L1 | This gene encodes a protein that homodimerizes and functions as a transcription factor which activates the expression of some key metabolic genes regulating cellular growth and nuclear genes required for respiration, heme biosynthesis, and mitochondrial DNA transcription and replication. The protein has also been associated with the regulation of neurite outgrowth. Alternative splicing results in multiple transcript variants. Confusion has occurred in bibliographic databases due to the shared symbol of NRF1 for this gene and for "nuclear factor (erythroid-derived 2)-like 1" which has an official symbol of NFE2L1. [provided by RefSeq, May 2014] |
|  | USF1 | This gene encodes a member of the basic helix-loop-helix leucine zipper family, and can function as a cellular transcription factor. The encoded protein can activate transcription through pyrimidine-rich initiator (Inr) elements and E-box motifs. This gene has been linked to familial combined hyperlipidemia (FCHL). Alternative splicing of this gene results in multiple transcript variants. A related pseudogene has been defined on chromosome 21. [provided by RefSeq, Feb 2013] |
|  | SNAI1 | The Drosophila embryonic protein snail is a zinc finger transcriptional repressor which downregulates the expression of ectodermal genes within the mesoderm. The nuclear protein encoded by this gene is structurally similar to the Drosophila snail protein, and is also thought to be critical for mesoderm formation in the developing embryo. At least two variants of a similar processed pseudogene have been found on chromosome 2. [provided by RefSeq, Jul 2008] |
|  | NKX25 | This gene encodes a homeobox-containing transcription factor. This transcription factor functions in heart formation and development. Mutations in this gene cause atrial septal defect with atrioventricular conduction defect, and also tetralogy of Fallot, which are both heart malformation diseases. Mutations in this gene can also cause congenital hypothyroidism non-goitrous type 5, a non-autoimmune condition. Alternative splicing results in multiple transcript variants. [provided by RefSeq, Oct 2009] |

**Supplementary Table 9.** **The TF annotations for top five enriched motifs in promoters for six different cell lines.** Although motif enrichment analysis is simple but the enriched motifs do represent some cell line-specific information, for example, enriched motifs in cancer-related cell lines might play a role in carcinogenesis.

| Cell lines | TF name | TF annotation |
| --- | --- | --- |
| K562 | PPARA | Peroxisome proliferators include hypolipidemic drugs, herbicides, leukotriene antagonists, and plasticizers; this term arises because they induce an increase in the size and number of peroxisomes. Peroxisomes are subcellular organelles found in plants and animals that contain enzymes for respiration and for cholesterol and lipid metabolism. The action obf peroxisome proliferators is thought to be mediated via specific receptors, called PPARs, which belong to the steroid hormone receptor superfamily. PPARs affect the expression of target genes involved in cell proliferation, cell differentiation and in immune and inflammation responses. Three closely related subtypes (alpha, beta/delta, and gamma) have been identified. This gene encodes the subtype PPAR-alpha, which is a nuclear transcription factor. Multiple alternatively spliced transcript variants have been described for this gene, although the full-length nature of only two has been determined. [provided by RefSeq, Jul 2008] |
|  | MAF | The protein encoded by this gene is a DNA-binding, leucine zipper-containing transcription factor that acts as a homodimer or as a heterodimer. Depending on the binding site and binding partner, the encoded protein can be a transcriptional activator or repressor. This protein plays a role in the regulation of several cellular processes, including embryonic lens fiber cell development, increased T-cell susceptibility to apoptosis, and chondrocyte terminal differentiation. Defects in this gene are a cause of juvenile-onset pulverulent cataract as well as congenital cerulean cataract 4 (CCA4). Two transcript variants encoding different isoforms have been found for this gene. [provided by RefSeq, Jan 2010] |
|  | THA | The protein encoded by this gene is a nuclear hormone receptor for triiodothyronine. It is one of the several receptors for thyroid hormone, and has been shown to mediate the biological activities of thyroid hormone. Knockout studies in mice suggest that the different receptors, while having certain extent of redundancy, may mediate different functions of thyroid hormone. Alternatively spliced transcript variants encoding distinct isoforms have been reported. [provided by RefSeq, Jul 2008] |
|  | GCR | This gene encodes glucocorticoid receptor, which can function both as a transcription factor that binds to glucocorticoid response elements in the promoters of glucocorticoid responsive genes to activate their transcription, and as a regulator of other transcription factors. This receptor is typically found in the cytoplasm, but upon ligand binding, is transported into the nucleus. It is involved in inflammatory responses, cellular proliferation, and differentiation in target tissues. Mutations in this gene are associated with generalized glucocorticoid resistance. Alternative splicing of this gene results in transcript variants encoding either the same or different isoforms. Additional isoforms resulting from the use of alternate in-frame translation initiation sites have also been described, and shown to be functional, displaying diverse cytoplasm-to-nucleus trafficking patterns and distinct transcriptional activities (PMID:15866175). [provided by RefSeq, Feb 2011] |
|  | COT2 | This gene encodes a member of the steroid thyroid hormone superfamily of nuclear receptors. The encoded protein is a ligand inducible transcription factor that is involved in the regulation of many different genes. Alternate splicing results in multiple transcript variants. [provided by RefSeq, Mar 2010] |
| IMR90 | LEF1 | This gene encodes a transcription factor belonging to a family of proteins that share homology with the high mobility group protein-1. The protein encoded by this gene can bind to a functionally important site in the T-cell receptor-alpha enhancer, thereby conferring maximal enhancer activity. This transcription factor is involved in the Wnt signaling pathway, and it may function in hair cell differentiation and follicle morphogenesis. Mutations in this gene have been found in somatic sebaceous tumors. This gene has also been linked to other cancers, including androgen-independent prostate cancer. Alternative splicing results in multiple transcript variants. [provided by RefSeq, Oct 2009] |
|  | SOX10 | This gene encodes a member of the SOX (SRY-related HMG-box) family of transcription factors involved in the regulation of embryonic development and in the determination of the cell fate. The encoded protein may act as a transcriptional activator after forming a protein complex with other proteins. This protein acts as a nucleocytoplasmic shuttle protein and is important for neural crest and peripheral nervous system development. Mutations in this gene are associated with Waardenburg-Shah and Waardenburg-Hirschsprung disease. [provided by RefSeq, Jul 2008] |
|  | MSX2 | This gene encodes a member of the muscle segment homeobox gene family. The encoded protein is a transcriptional repressor whose normal activity may establish a balance between survival and apoptosis of neural crest-derived cells required for proper craniofacial morphogenesis. The encoded protein may also have a role in promoting cell growth under certain conditions and may be an important target for the RAS signaling pathways. Mutations in this gene are associated with parietal foramina 1 and craniosynostosis type 2. [provided by RefSeq, Jul 2008] |
|  | KLF4 | This gene encodes a protein that belongs to the Kruppel family of transcription factors. The encoded zinc finger protein is required for normal development of the barrier function of skin. The encoded protein is thought to control the G1-to-S transition of the cell cycle following DNA damage by mediating the tumor suppressor gene p53. Mice lacking this gene have a normal appearance but lose weight rapidly, and die shortly after birth due to fluid evaporation resulting from compromised epidermal barrier function. Alternative splicing results in multiple transcript variants encoding different isoforms. [provided by RefSeq, Sep 2015] |
|  | SMAD3 | The protein encoded by this gene belongs to the SMAD, a family of proteins similar to the gene products of the Drosophila gene 'mothers against decapentaplegic' (Mad) and the C. elegans gene Sma. SMAD proteins are signal transducers and transcriptional modulators that mediate multiple signaling pathways. This protein functions as a transcriptional modulator activated by transforming growth factor-beta and is thought to play a role in the regulation of carcinogenesis. [provided by RefSeq, Apr 2009 |
| GM12878 | CXXC1 | This gene encodes a protein that functions as a transcriptional activator that binds specifically to non-methylated CpG motifs through its CXXC domain. The protein is a component of the SETD1 complex, regulates gene expression and is essential for vertebrate development. [provided by RefSeq, Sep 2015] |
|  | SOX15 | This gene encodes a member of the SOX (SRY-related HMG-box) family of transcription factors involved in the regulation of embryonic development and in the determination of the cell fate. The encoded protein may act as a transcriptional regulator after forming a protein complex with other proteins. [provided by RefSeq, Jul 2008] |
|  | PRRX1 | The DNA-associated protein encoded by this gene is a member of the paired family of homeobox proteins localized to the nucleus. The protein functions as a transcription co-activator, enhancing the DNA-binding activity of serum response factor, a protein required for the induction of genes by growth and differentiation factors. The protein regulates muscle creatine kinase, indicating a role in the establishment of diverse mesodermal muscle types. Alternative splicing yields two isoforms that differ in abundance and expression patterns. [provided by RefSeq, Jul 2008] |
|  | THB | The protein encoded by this gene is a nuclear hormone receptor for triiodothyronine. It is one of the several receptors for thyroid hormone, and has been shown to mediate the biological activities of thyroid hormone. Knockout studies in mice suggest that the different receptors, while having certain extent of redundancy, may mediate different functions of thyroid hormone. Mutations in this gene are known to be a cause of generalized thyroid hormone resistance (GTHR), a syndrome characterized by goiter and high levels of circulating thyroid hormone (T3-T4), with normal or slightly elevated thyroid stimulating hormone (TSH). Several alternatively spliced transcript variants encoding the same protein have been observed for this gene. [provided by RefSeq, Jul 2008] |
|  | MSX2 | This gene encodes a member of the muscle segment homeobox gene family. The encoded protein is a transcriptional repressor whose normal activity may establish a balance between survival and apoptosis of neural crest-derived cells required for proper craniofacial morphogenesis. The encoded protein may also have a role in promoting cell growth under certain conditions and may be an important target for the RAS signaling pathways. Mutations in this gene are associated with parietal foramina 1 and craniosynostosis type 2. [provided by RefSeq, Jul 2008] |
| HUVEC | MYB | This gene encodes a protein with three HTH DNA-binding domains that functions as a transcription regulator. This protein plays an essential role in the regulation of hematopoiesis. This gene may be aberrently expressed or rearranged or undergo translocation in leukemias and lymphomas, and is considered to be an oncogene. Alternative splicing results in multiple transcript variants. [provided by RefSeq, Jan 2016] |
|  | SOX15 | This gene encodes a member of the SOX (SRY-related HMG-box) family of transcription factors involved in the regulation of embryonic development and in the determination of the cell fate. The encoded protein may act as a transcriptional regulator after forming a protein complex with other proteins. [provided by RefSeq, Jul 2008] |
|  | CXXC1 | This gene encodes a protein that functions as a transcriptional activator that binds specifically to non-methylated CpG motifs through its CXXC domain. The protein is a component of the SETD1 complex, regulates gene expression and is essential for vertebrate development. [provided by RefSeq, Sep 2015] |
|  | GABP1+GABP2 | This gene encodes the GA-binding protein transcription factor, beta subunit. This protein forms a tetrameric complex with the alpha subunit, and stimulates transcription of target genes. The encoded protein may be involved in activation of cytochrome oxidase expression and nuclear control of mitochondrial function. The crystal structure of a similar protein in mouse has been resolved as a ternary protein complex. Multiple transcript variants encoding distinct isoforms have been identified for this gene. [provided by RefSeq, Jul 2008] |
|  | NKX21 | This gene encodes a protein initially identified as a thyroid-specific transcription factor. The encoded protein binds to the thyroglobulin promoter and regulates the expression of thyroid-specific genes but has also been shown to regulate the expression of genes involved in morphogenesis. Mutations and deletions in this gene are associated with benign hereditary chorea, choreoathetosis, congenital hypothyroidism, and neonatal respiratory distress, and may be associated with thyroid cancer. Multiple transcript variants encoding different isoforms have been found for this gene. This gene shares the symbol/alias 'TTF1' with another gene, transcription termination factor 1, which plays a role in ribosomal gene transcription. [provided by RefSeq, Feb 2014] |
| HeLa-S3 | DLX2 | Many vertebrate homeo box-containing genes have been identified on the basis of their sequence similarity with Drosophila developmental genes. Members of the Dlx gene family contain a homeobox that is related to that of Distal-less (Dll), a gene expressed in the head and limbs of the developing fruit fly. The Distal-less (Dlx) family of genes comprises at least 6 different members, DLX1-DLX6. The DLX proteins are postulated to play a role in forebrain and craniofacial development. This gene is located in a tail-to-tail configuration with another member of the gene family on the long arm of chromosome 2. [provided by RefSeq, Jul 2008] |
|  | DLX3 | Many vertebrate homeo box-containing genes have been identified on the basis of their sequence similarity with Drosophila developmental genes. Members of the Dlx gene family contain a homeobox that is related to that of Distal-less (Dll), a gene expressed in the head and limbs of the developing fruit fly. The Distal-less (Dlx) family of genes comprises at least 6 different members, DLX1-DLX6. Trichodentoosseous syndrome (TDO), an autosomal dominant condition, has been correlated with DLX3 gene mutation. This gene is located in a tail-to-tail configuration with another member of the gene family on the long arm of chromosome 17. Mutations in this gene have been associated with the autosomal dominant conditions trichodentoosseous syndrome and amelogenesis imperfecta with taurodontism. [provided by RefSeq, Jul 2008] |
|  | PRGR | This gene encodes a member of the steroid receptor superfamily. The encoded protein mediates the physiological effects of progesterone, which plays a central role in reproductive events associated with the establishment and maintenance of pregnancy. This gene uses two distinct promotors and translation start sites in the first exon to produce several transcript variants, both protein coding and non-protein coding. Two of the isoforms (A and B) are identical except for an additional 165 amino acids found in the N-terminus of isoform B and mediate their own response genes and physiologic effects with little overlap. [provided by RefSeq, Sep 2015] |
|  | PRRX2 | The DNA-associated protein encoded by this gene is a member of the paired family of homeobox proteins. Expression is localized to proliferating fetal fibroblasts and the developing dermal layer, with downregulated expression in adult skin. Increases in expression of this gene during fetal but not adult wound healing suggest a possible role in mechanisms that control mammalian dermal regeneration and prevent formation of scar response to wounding. The expression patterns provide evidence consistent with a role in fetal skin development and a possible role in cellular proliferation. [provided by RefSeq, Jul 2008] |
|  | HXB7 | This gene is a member of the Antp homeobox family and encodes a protein with a homeobox DNA-binding domain. It is included in a cluster of homeobox B genes located on chromosome 17. The encoded nuclear protein functions as a sequence-specific transcription factor that is involved in cell proliferation and differentiation. Increased expression of this gene is associated with some cases of melanoma and ovarian carcinoma. [provided by RefSeq, Jul 2008] |
| NHEK | KLF6 | This gene encodes a member of the Kruppel-like family of transcription factors. The zinc finger protein is a transcriptional activator, and functions as a tumor suppressor. Multiple transcript variants encoding different isoforms have been found for this gene, some of which are implicated in carcinogenesis. [provided by RefSeq, May 2009] |
|  | PAX2 | PAX2 encodes paired box gene 2, one of many human homologues of the Drosophila melanogaster gene prd. The central feature of this transcription factor gene family is the conserved DNA-binding paired box domain. PAX2 is believed to be a target of transcriptional supression by the tumor suppressor gene WT1. Mutations within PAX2 have been shown to result in optic nerve colobomas and renal hypoplasia. Alternative splicing of this gene results in multiple transcript variants. [provided by RefSeq, Dec 2014] |
|  | MAFB | The protein encoded by this gene is a basic leucine zipper (bZIP) transcription factor that plays an important role in the regulation of lineage-specific hematopoiesis. The encoded nuclear protein represses ETS1-mediated transcription of erythroid-specific genes in myeloid cells. This gene contains no introns. [provided by RefSeq, Jul 2008] |
|  | LEF1 | This gene encodes a transcription factor belonging to a family of proteins that share homology with the high mobility group protein-1. The protein encoded by this gene can bind to a functionally important site in the T-cell receptor-alpha enhancer, thereby conferring maximal enhancer activity. This transcription factor is involved in the Wnt signaling pathway, and it may function in hair cell differentiation and follicle morphogenesis. Mutations in this gene have been found in somatic sebaceous tumors. This gene has also been linked to other cancers, including androgen-independent prostate cancer. Alternative splicing results in multiple transcript variants. [provided by RefSeq, Oct 2009] |
|  | SOX15 | This gene encodes a member of the SOX (SRY-related HMG-box) family of transcription factors involved in the regulation of embryonic development and in the determination of the cell fate. The encoded protein may act as a transcriptional regulator after forming a protein complex with other proteins. [provided by RefSeq, Jul 2008] |

**Supplementary Figure 1. Length distribution of active enhancers in different cell lines.**

**Supplementary Figure 2. Length distribution of active promoters in different cell lines.**
